# Association of the lactate dehydrogenase-to-albumin ratio with all-cause and non-relapse mortality in patients with aGVHD after allogeneic HSCT: a single-center retrospective cohort study

**DOI:** 10.3389/fimmu.2026.1936503

**Published:** 2026-10-09

**Authors:** Gangping Li, Di Zhang, Fangfang Yuan, Minghui Li, Yongqi Wang, Jian Zhou, Yuewen Fu

**Affiliations:** 1 Department of Hematology, The Affiliated Cancer Hospital of Zhengzhou University & Henan Cancer Hospital, Zhengzhou, China; 2 Department of Medical Records Management, The Affiliated Cancer Hospital of Zhengzhou University & Henan Cancer Hospital, Zhengzhou, China

**Keywords:** acute graft-versus-host disease, allogeneic hematopoietic stem cell transplantation, biomarker, lactate dehydrogenase to albumin ratio, prognosis

## Abstract

**Background:**

Acute graft-versus-host disease (aGVHD) remains a leading cause of non-relapse mortality after allogeneic hematopoietic stem cell transplantation (allo-HSCT). The lactate dehydrogenase-to-albumin ratio (LAR), integrating metabolic stress and nutritional reserve, has demonstrated prognostic value across malignancies but has not been investigated in aGVHD. We evaluated the association between LAR measured at aGVHD diagnosis and both all-cause mortality (ACM) and non-relapse mortality (NRM).

**Methods:**

This retrospective cohort study enrolled 109 consecutive allo-HSCT recipients who developed aGVHD at Henan Cancer Hospital (2019–2024). Patients were stratified by LAR tertiles (T1:4.37–8.84;T2:8.84–16.02;T3:16.02–178.02).Natural-log-transformed LAR (ln-LAR) was analyzed using Cox proportional hazards models with three hierarchical adjustments: Model 1 (age, sex), Model 2 (+transplantation covariates), and Model 3 (+post-transplant clinical covariates). Restricted cubic spline regression and two-piecewise threshold analyses characterized dose-response relationships. Subgroup, interaction, and sensitivity analyses were performed to evaluate the robustness.

**Results:**

Over a median follow-up of 30 months, 69 ACM (63.3%) and 66 NRM (60.6%) events occurred among 109 patients (mean age 29.4 ± 15.3 years, 62.4% male; 51 grade I–II, 58 grade III–IV aGVHD). Continuous ln-LAR independently predicted ACM (Model 3: HR 1.73, 95% CI 1.07–2.77, *P* = 0.024) and NRM (Model 3: HR 1.69, 95% CI 1.03–2.77, *P* = 0.038). The highest tertile (T3) conferred approximately three-fold higher risk versus T1 (ACM: HR 2.96, 95% CI 1.27–6.90, *P* = 0.012; NRM: HR 3.25, 95% CI 1.36–7.74, *P* = 0.008). RCS demonstrated nonlinear inverted-L patterns (nonlinearity *P* = 0.006 for ACM, *P* = 0.009 for NRM) with threshold effects at LAR≈53 and ≈19, respectively. Subgroup and interaction analyses revealed no significant modifiers (all *P* for interaction >0.05), and sensitivity analyses excluding early deaths yielded consistent results with amplified effect sizes.

**Conclusion:**

Elevated LAR at aGVHD diagnosis was independently associated with higher all-cause and non-relapse mortality in this cohort. These findings are hypothesis-generating; the clinical utility of LAR requires external validation in independent cohorts.

## Introduction

Allogeneic hematopoietic stem cell transplantation (allo-HSCT) constitutes a potentially curative therapeutic modality for a spectrum of hematologic malignancies, bone marrow failure syndromes, and selected non-malignant disorders (1–3). Substantial advances in donor selection, conditioning regimens, graft-versus-host disease (GVHD) prophylaxis, and supportive care have progressively expanded the applicability of this procedure to older and medically complex patient population (4, 5). Despite these refinements, transplant-related morbidity and mortality remain substantial, with acute graft-versus-host disease (aGVHD) representing one of the most formidable obstacles to favorable outcomes (6, 7). aGVHD, primarily mediated by donor T-cell recognition of host alloantigens, triggers a complex inflammatory cascade culminating in target organ damage involving the skin, liver, and gastrointestinal tract (6). The clinical trajectory of aGVHD is highly variable; whereas some patients respond satisfactorily to first-line corticosteroid therapy, a substantial proportion progress to steroid-refractory disease, which portends an exceedingly poor prognosis with mortality rates exceeding 50% (8, 9).

Accurate risk stratification at the onset of aGVHD is critical for guiding treatment intensity, facilitating early intervention, and ultimately improving survival. However, current prognostic assessment relies predominantly on clinical parameters—including aGVHD grade, organ involvement, and response to initial therapy—that capture only a fraction of the underlying biological heterogeneity (10, 11). In response to this limitation, considerable investigative effort has been directed toward identifying circulating biomarkers that reflect the pathophysiologic processes driving aGVHD. The Mount Sinai Acute GVHD International Consortium (MAGIC) has established a panel comprising suppression of tumorigenicity 2 (ST2) and regenerating islet-derived 3-α (REG3α), which robustly predicts treatment response and non-relapse mortality (12, 13). Additional candidate biomarkers, including tumor necrosis factor receptor 1 (TNFR1), elafin, and interleukin-2 receptor α, have demonstrated association with aGVHD severity and outcomes (14, 15). Despite these advances, many of these assays require specialized platforms and are not routinely available in most clinical laboratories, highlighting the need for accessible, low-cost biomarkers that can be derived from standard laboratory data.

Serum lactate dehydrogenase (LDH) is a ubiquitous intracellular enzyme catalyzing the interconversion of lactate and pyruvate; its elevation in the circulation reliably reflects cellular turnover, tissue hypoxia, and metabolic stress (16, 17). A comprehensive meta-analysis of 68 studies encompassing 31,857 patients with solid tumors confirmed that elevated LDH is associated with a 48% increased risk of death (HR 1.48, 95% CI 1.43–1.53), establishing LDH as a robust prognostic biomarker across multiple malignancies (16). Elevated LDH has also been consistently associated with adverse outcomes in the peri-transplant setting (18–20). Specifically, pre-transplant hyper-LDH predicts inferior overall survival and higher relapse incidence in patients with acute myeloid leukemia and lymphoma undergoing allo-HSCT (21, 22). Serum albumin, conversely, serves as a proxy for nutritional reserve and systemic inflammatory burden; hypoalbuminemia has been linked to increased non-relapse mortality in patients with lower gastrointestinal aGVHD (23). Composite inflammatory and nutritional indices, such as the C-reactive protein-to-albumin ratio, have demonstrated prognostic utility in allo-HSCT recipients (24, 25). The lactate dehydrogenase to albumin ratio (LAR), which integrates the metabolic dimension captured by LDH with the nutritional-inflammatory dimension reflected by albumin, has emerged as a promising prognostic biomarker across multiple solid tumor types (26–29). A recent meta-analysis encompassing over 8,000 patients confirmed that elevated pretreatment LAR is significantly associated with inferior overall survival (HR 2.02, 95% CI 1.74–2.34) and progression-free survival in malignancies (30). However, the prognostic value of LAR in the specific context of aGVHD following allo-HSCT has not been systematically investigated.

Accordingly, this study aimed to evaluate the association between LAR, measured at the time of aGVHD diagnosis, and all-cause mortality (ACM) and non-relapse mortality (NRM) in patients undergoing allo-HSCT.

The remainder of this article is structured as follows. Section 2 (Methods) describes the study population, the definitions of LAR and the outcomes, and the statistical analyses, including restricted cubic spline and threshold analyses. Section 3 (Results) reports the association of LAR with all-cause and non-relapse mortality across tertiles and strata. Sections 4 and 5 present the discussion of the findings in the context of existing evidence, the study limitations, and our conclusions.

## Methods

### Patients and study design

This retrospective cohort study consecutively enrolled patients undergoing allo-HSCT at Henan Cancer Hospital between January 2019 and December 2024. Within this transplant cohort, patients who developed aGVHD were identified according to established clinical and histopathological diagnostic criteria (1). Exclusion criteria comprised: (1) had active, uncontrolled infection at aGVHD onset; (2) incomplete LDH or albumin measurements within 48 hours of aGVHD diagnosis; (3) loss to follow-up prior to study completion. The final analytical cohort comprised 109 patients. The study protocol was approved by the Research Ethics Committee of Henan Cancer Hospital (registration number: 2026-304-001), and all participants provided written informed consent. All procedures were conducted in accordance with the Declaration of Helsinki.

### Conditioning regimens and GVHD prophylaxis

Transplantation conditioning intensity was classified as myeloablative conditioning (MAC) or reduced-intensity conditioning (RIC) according to established consensus criteria (29–31). MAC regimens included busulfan combined with cyclophosphamide (Bu/Cy) or total body irradiation combined with cyclophosphamide (TBI/Cy). RIC regimens predominantly comprised fludarabine combined with busulfan (Flu/Bu, busulfan ≤6.4 mg/kg) or fludarabine combined with melphalan (Flu/Mel). For patients receiving grafts from unrelated or haploidentical donors, conditioning incorporated anti-human thymocyte immunoglobulin (ATG, 2.5 mg/kg/day for 3–4 days) for *in vivo* T-cell depletion, whereas sibling donor transplants proceeded without ATG. GVHD prophylaxis consisted of a calcineurin inhibitor (cyclosporine A, targeted trough 200–400 ng/mL, or tacrolimus, targeted trough 5–15 ng/mL) combined with methotrexate (15 mg/m² on day +1, 10 mg/m² on days +3, +6, and +11) and mycophenolate mofetil (MMF, 500–1000 mg every 12 hours from day −3 to day +30, tapered thereafter). Stem cell sources included peripheral blood stem cells (PBSC), bone marrow (BM), or a combination thereof.

### Supportive care

All transplant recipients received standardized supportive care according to institutional protocols. Antiviral prophylaxis consisted of ganciclovir (5 mg/kg intravenously every 12 hours) or valganciclovir, initiated upon neutrophil engraftment, with preemptive monitoring for cytomegalovirus (CMV) and Epstein-Barr virus (EBV) viremia by quantitative polymerase chain reaction twice weekly through day +100, followed by weekly monitoring thereafter. Antifungal prophylaxis comprised posaconazole suspension (200 mg three times daily) or voriconazole (200 mg twice daily, with therapeutic drug monitoring). Pneumocystis jirovecii prophylaxis was administered with sulfamethoxazole-trimethoprim (800/160 mg twice daily, three times weekly) from engraftment until immunosuppression discontinuation. Phenytoin (5 mg/kg/day) was administered for seizure prophylaxis during busulfan conditioning. Mesna (equivalent to 80–120% of cyclophosphamide dose) was employed for hemorrhagic cystitis prevention. Ursodeoxycholic acid (300 mg twice daily) was administered for hepatobiliary protection. Blood products were leukoreduced, irradiated, and CMV-seronegative where indicated. Febrile neutropenia was managed empirically with broad-spectrum antibiotics per institutional guidelines, with subsequent de-escalation guided by microbiologic culture results.

### LAR measurement and data collection

The lactate dehydrogenase to albumin ratio (LAR) was calculated as serum LDH activity (U/L) divided by serum albumin concentration (g/L) (26–29), measured within 48 hours of aGVHD diagnosis. For regression analyses, LAR values were natural-log transformed (ln-LAR) to reduce skewness and improve model fit. Serum LDH activity was determined using the lactate-to-pyruvate enzymatic reduction method on a Beckman Coulter AU5800 clinical chemistry analyzer (Beckman Coulter, Brea, CA, USA). Serum albumin was quantified using the bromocresol green dye-binding method on the same platform. Comprehensive demographic, clinical, and laboratory parameters were abstracted from electronic medical records, including: age, sex, indication for HSCT, stem cell source, transplant type (matched sibling, unrelated, haploidentical), conditioning regimen intensity (MAC vs RIC), ABO compatibility, mononuclear cell (MNC) count, CD34+ cell count, interval from transplantation to aGVHD diagnosis, aGVHD grade (I–II vs III–IV), neutrophil engraftment time, CMV viremia, EBV viremia, complete blood count, liver and renal function parameters, and infectious complications.

### Outcomes

The primary endpoint was all-cause mortality (ACM), defined as death from any cause during the follow-up period. The secondary endpoint was non-relapse mortality (NRM), defined as death without preceding disease relapse or progression. Follow-up duration was calculated from the date of aGVHD diagnosis to death or last contact, with administrative censoring on December 31, 2024. Survival status was ascertained through outpatient clinic records, hospital information systems, and telephone follow-up.

### Statistical analysis

Normality of variable distributions was evaluated through histogram inspection, quantile-quantile plots, and the Kolmogorov-Smirnov test. Continuous variables following a normal distribution were reported as means with standard deviations (mean ± SD), whereas non-normally distributed variables were summarized as medians with interquartile ranges (median [IQR]). Categorical variables were expressed as frequencies with percentages. Between-group comparisons were performed using analysis of variance (ANOVA) for normally distributed continuous variables, the Kruskal-Wallis test for skewed continuous variables, and Pearson’s chi-square or Fisher’s exact tests for categorical variables, as appropriate.

The association between natural-log-transformed LAR (ln-LAR) and mortality endpoints was quantified using Cox proportional hazards regression models, with results reported as hazard ratios (HR) accompanied by 95% confidence intervals (CI). The proportional hazards assumption was verified through log-log survival plots and examination of time-dependent covariate interactions. Missing data at the aGVHD diagnosis time point (LDH, albumin, and other covariates) were assessed; all 109 included patients had complete LDH and albumin values required to compute LAR, as patients with incomplete measurements were excluded before enrollment. No imputation was performed; complete-case analysis was applied in all primary models, and the numbers of patients and events in each model are reported in the corresponding table. Three sequential Cox regression models were developed: Model 1 adjusted for demographic factors (age, sex); Model 2 incorporated Model 1 covariates plus transplantation parameters (indication for HSCT, stem cell source, transplant type, conditioning regimen, interval from transplantation to aGVHD diagnosis, ABO compatibility, MNC count, and CD34+ cell count); Model 3 expanded Model 2 with post-transplant clinical covariates (neutrophil engraftment time, CMV viremia, EBV viremia, white blood cell count, hemoglobin, platelet count, total bilirubin, creatinine, pulmonary infection, intestinal infection, febrile neutropenia).

Restricted cubic spline (RCS) regression with three knots positioned at the 10th, 50th, and 90th percentiles characterized the dose-response profile. A two-piecewise Cox regression model identified potential threshold effects, with breakpoints determined by maximum likelihood estimation. Prior to the two-piecewise Cox breakpoint estimation, natural-log-transformed LAR values were screened with Grubbs’ test (two-sided, α = 0.05). One outlier was identified and excluded for the ACM analysis (N = 108) and one for the NRM analysis (N = 108), preventing extreme LAR values from dominating the segmented fit; these patients remained in the primary Cox analyses. Kaplan-Meier survival curves stratified by LAR tertiles were generated, and between-group survival differences were assessed using the log-rank test. Subgroup and interaction analyses were performed across clinically relevant strata. Sensitivity analyses excluding patients with survival duration <3 months were conducted to evaluate the robustness of the primary findings. All statistical analyses were performed using R software version 4.3.2 (R Foundation for Statistical Computing, Vienna, Austria) and Free Statistics analysis platform. A two-sided P value <0.05 was considered statistically significant.

## Results

### Baseline characteristics

The participant selection pathway is depicted in **Figure 1**. Among 521 consecutive patients who underwent allo-HSCT during the study period, 116 developed aGVHD. Following the exclusion of 7 patients due to active/uncontrolled infection at aGVHD onset or incomplete LDH/albumin data or loss to follow-up, 109 patients who developed aGVHD after allo-HSCT were retained for the final analytical cohort (**Figure 1**). **Table 1** delineates the baseline profiles of the 109 participants grouped by LAR tertiles (T1–T3). The cohort exhibited a mean age of 29.4 ± 15.3 years, with a male predominance of 62.4%. Across LAR strata, several variables differed significantly. The proportion of grade III–IV aGVHD increased progressively from T1 (22.2%) to T2 (55.6%) to T3 (81.1%) (*P* < 0.001). Creatinine and blood urea nitrogen levels were significantly elevated in the highest tertile (*P* = 0.009 and *P* = 0.013, respectively). Aspartate aminotransferase increased across tertiles (*P* < 0.001). CMV viremia was more frequent in higher LAR groups (*P* = 0.002). Notably, patients in T1 had a shorter interval from transplantation to aGVHD diagnosis (median 26.0 vs 75.5–76.0 days, *P* < 0.001). Older age was observed in T1 compared with T2–T3 (*P* = 0.031). No significant differences were observed across LAR groups with respect to sex, indication for HSCT, stem cell source, transplant type, conditioning regimen intensity, ABO compatibility, neutrophil engraftment time, EBV viremia, white blood cell count, hemoglobin, platelet count, total bilirubin, or alanine aminotransferase (all *P*>0.05).

**Figure 1:**
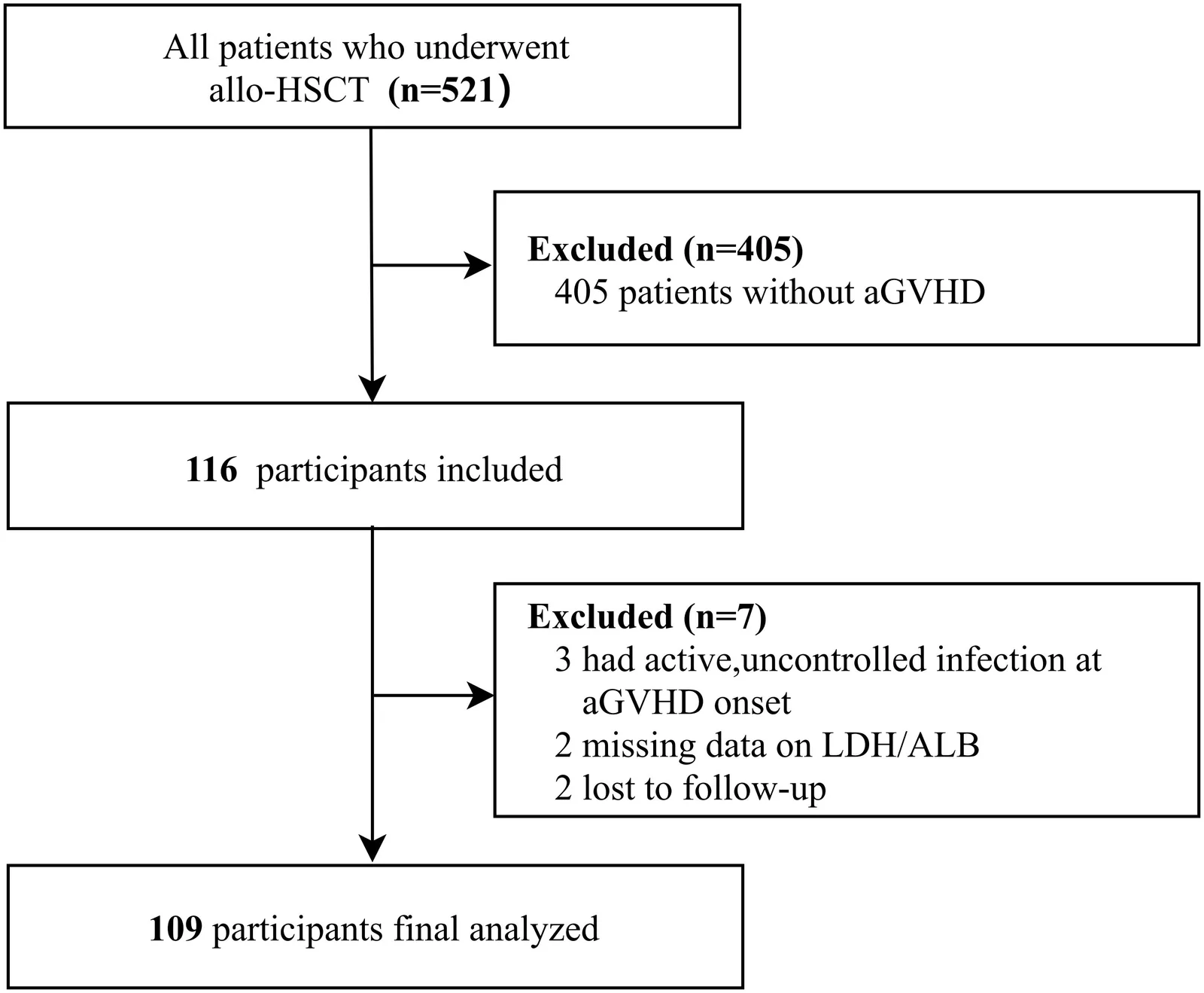
Study flow diagram of cohort derivation. Among 521 consecutive patients who underwent allogeneic hematopoietic stem cell transplantation (allo-HSCT) between January 2019 and December 2024, 116 developed acute graft-versus-host disease (aGVHD). After excluding 7 patients due to incomplete lactate dehydrogenase (LDH) or albumin measurements within 48 hours of aGVHD diagnosis or loss to follow-up, 109 patients were included in the final analytical cohort.

**Table 1:** The baseline characteristics and demographic characteristics of the study population by categories of LAR.

Characteristic	LAR				
	Total (n = 109)	1 (n = 36)	2 (n = 36)	3 (n = 37)	*p*-Value
Age, Mean ± SD	29.4 ± 15.3	34.7 ± 14.9	25.6 ± 14.7	28.1 ± 15.1	0.031
Sex, n (%)					0.525
Male	68 (62.4)	22 (61.1)	25 (69.4)	21 (56.8)	
Female	41 (37.6)	14 (38.9)	11 (30.6)	16 (43.2)	
Indication for HSCT, n(%)					0.866
AML	29 (26.6)	13 (36.1)	9 (25)	7 (18.9)	
ALL	34 (31.2)	9 (25)	12 (33.3)	13 (35.1)	
MDS	16 (14.7)	4 (11.1)	5 (13.9)	7 (18.9)	
SAA	20 (18.3)	7 (19.4)	6 (16.7)	7 (18.9)	
Others	10 ( 9.2)	3 (8.3)	4 (11.1)	3 (8.1)	
Stem cell sources, *n* (%)					0.47
Bone marrow	103 (94.5)	33 (91.7)	34 (94.4)	36 (97.3)	
Peripheral blood	5 ( 4.6)	3 (8.3)	1 (2.8)	1 (2.7)	
Bone marrow + Peripheral blood	1 ( 0.9)	0 (0)	1 (2.8)	0 (0)	
Type of transplantation, *n* (%)					0.799
Unrelated match	50 (45.9)	15 (41.7)	18 (50)	17 (45.9)	
HLA match related	35 (32.1)	13 (36.1)	12 (33.3)	10 (27)	
Haplo-identical related	24 (22.0)	8 (22.2)	6 (16.7)	10 (27)	
Conditioning regimen, *n* (%)					0.476
MAC	56 (51.4)	20 (55.6)	20 (55.6)	16 (43.2)	
RIC	53 (48.6)	16 (44.4)	16 (44.4)	21 (56.8)	
ABO match, *n* (%)					0.684
Matched	42 (38.5)	12 (33.3)	14 (38.9)	16 (43.2)	
Unmatched	67 (61.5)	24 (66.7)	22 (61.1)	21 (56.8)	
Disease status before HSCT, n (%) ^a^					0.711
CR	70 (64.2)	23 (63.9)	26 (72.2)	21 (56.8)	
PR or NR	18 (16.5)	6 (16.7)	4 (11.1)	8 (21.6)	
MRD before HSCT, n (%) ^a,b^					0.483
Positive	68 (62.4)	22 (61.1)	26 (72.2)	20 (54.1)	
Negative	21 (19.3)	7 (19.4)	4 (11.1)	10 (27)	
MNC count, ×10^8^/Kg, Mean ± SD	13.4 ± 6.5	13.4 ± 7.8	12.8 ± 5.0	13.9 ± 6.7	0.752
CD34^+^ cells count, ×10^6^/Kg, Mean ± SD	6.8 ± 4.2	6.0 ± 3.9	6.7 ± 2.7	7.6 ± 5.4	0.262
Neutrophil engraftment time, days, Mean ± SD	12.5 ± 2.3	12.4 ± 3.1	12.7 ± 1.8	12.5 ± 1.9	0.84
Days from transplantation to aGVHD diagnosis	49.0 (20.0, 100.0)	26.0 (12.8, 45.8)	75.5 (37.8, 121.0)	76.0 (32.0, 132.0)	< 0.001
CMV viremia, n (%)	24 (22.0)	1 (2.8)	10 (27.8)	13 (35.1)	0.002
EBV viremia, n (%)	18 (16.5)	6 (16.7)	7 (19.4)	5 (13.5)	0.792
Pulmonary infection, n (%)	81 (74.3)	22 (61.1)	30 (83.3)	29 (78.4)	0.076
Intestinal infection, n (%)	58 (53.2)	15 (41.7)	19 (52.8)	24 (64.9)	0.139
Febrile neutropenia, n (%)	31 (28.4)	6 (16.7)	12 (33.3)	13 (35.1)	0.158
White blood cells, ×10^9^/L, Median (IQR)	2.5 (1.1, 4.4)	2.5 (0.8, 3.9)	2.5 (1.8, 4.4)	2.2 (1.0, 3.9)	0.356
Hemoglobin, g/L, Mean ± SD	87.2 ± 21.0	91.4 ± 22.0	87.6 ± 19.2	82.6 ± 21.2	0.199
Platelets, ×10^9^/L, Median (IQR)	37.0 (22.0, 72.0)	42.0 (23.0, 73.0)	46.0 (23.8, 83.8)	30.0 (17.0, 48.0)	0.21
Total bilirubin, (umol/L), Median (IQR)	17.8 (11.2, 33.4)	19.4 (10.7, 32.1)	20.6 (12.3, 37.7)	15.9 (10.8, 33.4)	0.519
Alanine Aminotransferase, (U/L), Median (IQR)	23.0 (15.0, 64.0)	17.0 (9.0, 44.5)	28.0 (16.0, 86.0)	29.0 (19.0, 62.0)	0.055
Aspartate Aminotransferase, (U/L),Median (IQR)	35.0 (20.0, 62.0)	20.0 (12.8, 34.5)	39.0 (24.0, 79.8)	46.0 (29.0, 68.0)	< 0.001
Blood Urea Nitrogen, (mmol/L), Median (IQR)	6.6 (4.2, 10.9)	5.8 (3.6, 9.8)	5.7 (3.7, 9.4)	9.0 (5.5, 14.2)	0.013
Creatinine, (μmol/L), Median (IQR)	51.0 (35.0, 84.0)	49.0 (35.8, 62.2)	44.0 (29.0, 63.2)	70.0 (48.0, 106.0)	0.009
Status, n (%)					< 0.001
Alive	40 (36.7)	22 (61.1)	14 (38.9)	4 (10.8)	
All-cause mortality	69 (63.3)	14 (38.9)	22 (61.1)	33 (89.2)	
NRM	66 (60.6)	14 (38.9)	21 (58.3)	31 (83.8)	
Disease relapse/progression	3 ( 2.8)	0 (0)	1 (2.8)	2 (5.4)	
Time, months, Median (IQR)					
from stem cell infusion to death from any cause	6.0 (3.0, 18.0)	14.0 (5.0, 23.0)	6.5 (3.8, 12.8)	5.0 (3.0, 8.0)	0.036
from stem cell infusion to relapse/progression or death	6.0 (3.0, 16.0)	14.0 (5.0, 23.0)	6.5 (3.8, 12.8)	5.0 (3.0, 8.0)	0.033
aGVHD , n (%)					< 0.001
Grade I-II	51 (46.8)	28 (77.8)	16 (44.4)	7 (18.9)	
Grade III-IV	58 (53.2)	8 (22.2)	20 (55.6)	30 (81.1)	

^a^ Indicates that patients with n=20 SAA were excluded from the MRD and disease status statistics before HSCT;

^b^ MRD was measured by flow cytometry before HSCT and MRD levels≥10^−4^ was defined as positive;

LAR, Lactate Dehydrogenase to Albumin Ratio; T1, LAR (4.37-8.84); T2, LAR (8.84-16.02); T3, LAR (16.02-178.02); SD, Standard Deviation; IQR, Interquartile Range; AML, acute myelocytic leukemia; ALL, acute lymphocytic leukemia; MDS, myelodysplastic syndrome; SAA, severe aplastic anemia; MAC, myeloablative conditioning; RIC, reduced-intensity conditioning; CR, complete remission; PR, partial remission; NR, non-remission; MNC, mononuclear cell count; MRD; minimal residual disease; CMV, cytomegalovirus; EBV, Epstein-Barr Virus; aGVHD, acute graft-versus-host disease.

### Restricted cubic spline analysis of the LAR-mortality dose-response relationship

Restricted cubic spline regression revealed a nonlinear inverted-L dose-response relationship between LAR and both mortality endpoints (**Figure 2**). For ACM (**Figure 2A**), the hazard function increased steeply at lower LAR values and plateaued beyond a threshold near LAR≈53, with a significant nonlinearity test (*P* = 0.006). For NRM (**Figure 2B**), a similar inverted-L pattern was observed with a more pronounced inflection point at LAR≈19 (nonlinearity *P* = 0.009). The two-piecewise threshold analyses confirmed these patterns: below the breakpoint, each unit increment in LAR conferred a 4.1% increase in ACM hazard (HR 1.041, 95% CI 1.003–1.080, P = 0.034) and a 13.6% increase in NRM hazard (HR 1136, 95% CI 1.055–1.223, *P*<0.001); above the respective thresholds, the associations were attenuated and non-significant (**Supplementary Tables 1**, **2**). These results confirm the inverted-L pattern with a saturation effect for both mortality endpoints.

**Figure 2:**
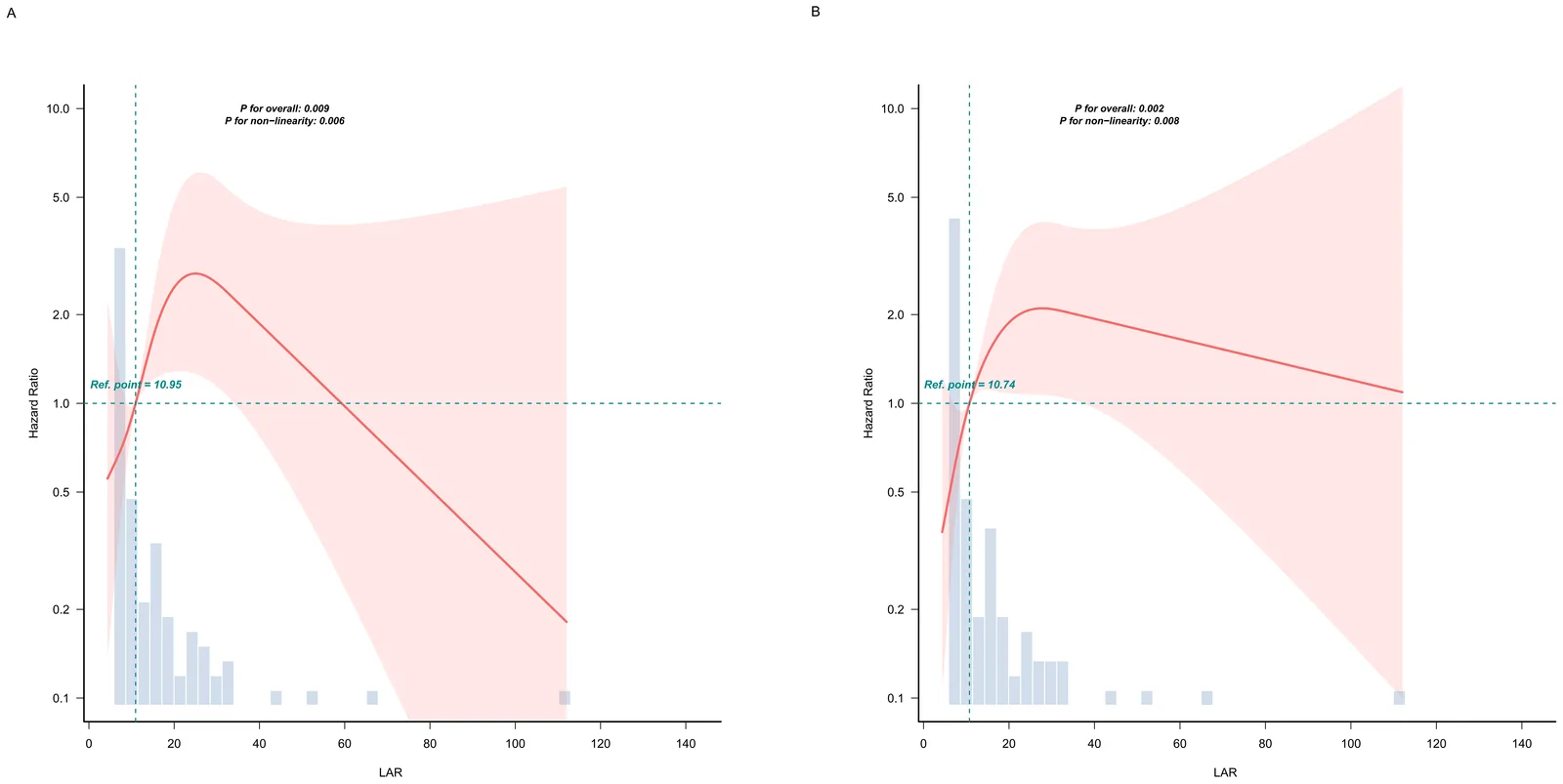
Restricted cubic spline (RCS) regression of the dose-response relationship between the lactate dehydrogenase-to-albumin ratio (LAR) and mortality. RCS curves with three knots at the 10th, 50th, and 90th percentiles were adjusted for age, sex, transplantation covariates, and post-transplant clinical covariates (Model 3). Solid lines represent hazard ratios (HR); shaded areas indicate 95% confidence intervals. Dashed vertical lines mark the estimated breakpoints from two-piecewise threshold analysis. **(A)** Association between LAR and all-cause mortality (ACM). Nonlinearity *P*=0.006; threshold at LAR≈53. **(B)** Association between LAR and non-relapse mortality (NRM). Nonlinearity *P* =0.009; threshold at LAR≈37.

### Associations between LAR and prognosis

**Table 2** reports the multivariable Cox regression findings assessing LAR in relation to ACM and NRM. With respect to ACM (69 events among 109 participants), log-transformed LAR (LDHALBln), when evaluated as a continuous variable, exhibited a significant positive association in the unadjusted framework (crude HR 1.97, 95% CI 1.44–2.70, *P* < 0.001). This association remained significant after progressive adjustment: Model 1 (adjusted HR 2.15, 95% CI 1.54–3.01, *P* < 0.001), Model 2 (adjusted HR 2.09, 95% CI 1.38–3.17, *P* = 0.001), and Model 3 (adjusted HR 1.73, 95% CI 1.07–2.77, *P* = 0.024). When evaluated categorically by tertiles, the highest LAR tertile (T3) was associated with a significantly elevated ACM risk relative to T1 across all models (Model 3: adjusted HR 2.96, 95% CI 1.27–2.90, *P* = 0.012). The intermediate tertile (T2) did not reach statistical significance in fully adjusted models (Model 3: adjusted HR 1.39, 95% CI 0.60–3.20, *P* = 0.441), though the trend test across tertiles remained significant (*P* = 0.006), supporting a graded dose-response pattern.

**Table 2:** Association between LAR and ACM and NRM in patients with aGVHD following allo-HSCT.

Characteristic	Total, n (%)	Event, n (%)	Crude model		Model 1		Model 2		Model 3	
			HR (95%CI)	*p*-Value	HR (95%CI)	*p*-Value	HR (95%CI)	*p*-Value	HR (95%CI)	*p*-Value
ACM										
ln LAR	109	69 (63.3)	1.97 (1.44~2.70)	<0.001	2.15 (1.54~3.01)	<0.001	2.09 (1.38~3.17)	0.001	1.73 (1.07~2.77)	0.024
LAR category										
T1	36	16 (44.4)	1(Ref)		1(Ref)		1(Ref)		1(Ref)	
T2	36	25 (69.4)	1.81 (0.92~3.57)	0.086	1.98 (0.98~4)	0.058	1.71 (0.81~3.62)	0.16	1.39 (0.60~3.20)	0.441
T3	37	28 (75.7)	3.62 (1.91~6.87)	<0.001	4.07 (2.1~7.92)	<0.001	3.6 (1.68~7.71)	0.001	2.96 (1.27~6.90)	0.012
Trend test				<0.001		<0.001		0.001		0.006
NRM										
ln LAR	109	66 (60.6)	1.85 (1.33~2.56)	<0.001	2.07 (1.46~2.94)	<0.001	2.06 (1.34~3.17)	0.001	1.69 (1.03~2.77)	0.038
LAR category										
T1	36	14 (38.9)	1(Ref)		1(Ref)		1(Ref)		1(Ref)	
T2	36	21 (58.3)	1.78 (0.9~3.55)	0.1	2.01 (0.98~4.12)	0.056	1.80 (0.83~3.86)	0.134	1.32 (0.56~3.13)	0.530
T3	37	31 (83.8)	3.43 (1.8~6.57)	<0.001	4.06 (2.06~8)	<0.001	4.05 (1.85~8.88)	<0.001	3.25 (1.36~7.74)	0.008
Trend test				<0.001		<0.001		<0.001		0.004

LAR, Lactate Dehydrogenase to Albumin Ratio; T1, LAR (4.37-8.84); T2, LAR (8.84-16.02); T3, LAR (16.02-178.02); ACM, all-cause mortality; NRM, non-relapse mortality; aGVHD, acute graft-versus-host disease; allo-HSCT, allogeneic hematopoietic stem cell transplantation; HR, Hazard Ratio; CI, Confidence Interval; Ref, reference.

Model 1: Adjusted for Age and Sex;

Model2: Adjusted for Model1 and Indication for HSCT, Stem cell sources, Type of transplantation, Conditioning regimen, Days from transplantation to diagnosis, ABO match, MNC count, CD34^+^ cells count;

Model3: Adjusted for Model2 and Neutrophil engraftment time, CMV viremia, EBV viremia, White blood cells, Hemoglobin and Platelets, *Total bilirubin*, Creatinine, Pulmonary infection, Intestinal infection, Febrile neutropenia, aGVHD.

Parallel analyses for NRM (66 events) yielded concordant patterns. Continuous log-LAR remained significantly linked to NRM after full adjustment (crude HR 1.85, 95% CI 1.33–2.56, *P* < 0.001; Model 3 adjusted HR 1.69, 95% CI 1.03–2.77, *P* = 0.038). Tertile comparisons demonstrated that T3 conveyed roughly a three-fold higher NRM hazard compared with T1 in fully adjusted models (Model 3: adjusted HR 3.25, 95% CI 1.36–7.74, *P* = 0.008). The trend test across tertiles was significant (*P* = 0.004). T2 showed a non-significant elevated risk (Model 3: adjusted HR 1.32, 95% CI 0.56–3.13, *P* = 0.530), consistent with the pattern observed for ACM.

Kaplan-Meier survival functions stratified by LAR classification are displayed in **Figure 3**. The log-rank test revealed highly significant divergence among the three tertiles for both ACM (*P* < 0.001) and NRM (*P* < 0.001). Regarding ACM (**Figure 3A**), median survival times were 14.0, 6.5, and 5.0 months for T1, T2, and T3, respectively (*P* < 0.001). For NRM (**Figure 3B**), the corresponding median survival estimates were 17.0, 7.0, and 5.0 months across ascending tertiles (*P* < 0.001). The survival curves for T1 and T2 diverged progressively over follow-up, whereas T3 exhibited the steepest early decline, underscoring the particularly adverse prognosis associated with the highest LAR stratum.

**Figure 3:**
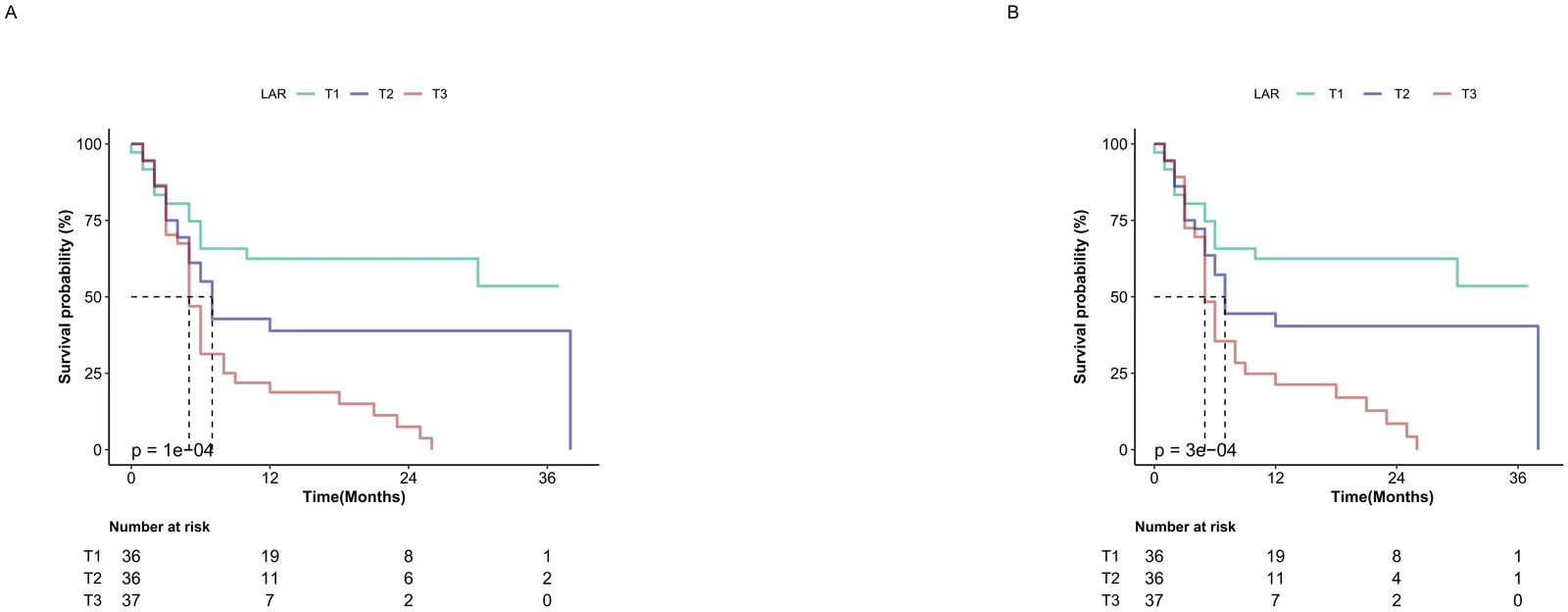
Kaplan-Meier survival curves stratified by LAR tertiles (T1: 4.37–8.84; T2: 8.84–16.02; T3: 16.02–178.02). Between-group differences were assessed by the log-rank test. **(A)** All-cause mortality (ACM). Median survival: T1=14.0 months, T2=6.5 months, T3=5.0 months (*P*<0.001). **(B)** Non-relapse mortality (NRM). Median survival: T1=17.0 months, T2=7.0 months, T3=5.0 months (*P*<0.001). n = 109 (T1: 36, T2: 36, T3: 37).

### Subgroup and interaction analyses

Subgroup and interaction analyses were performed to evaluate whether the LAR–mortality relationship varied across clinically relevant patient categories (**Figure 4**). The forest plot illustrates adjusted HRs for the T3/T2 versus T1 comparison across seven predefined strata. Although the point estimates varied in magnitude, all subgroups demonstrated a consistent direction of effect favoring lower LAR. No statistically significant interactions were detected for age (<40 vs ≥40 years), sex, ABO match, conditioning regimen, type of transplantation, CMV viremia and CD34 (all P>0.05). These results affirm the stability and generalizability of the LAR–mortality association across diverse clinical contexts.

**Figure 4:**
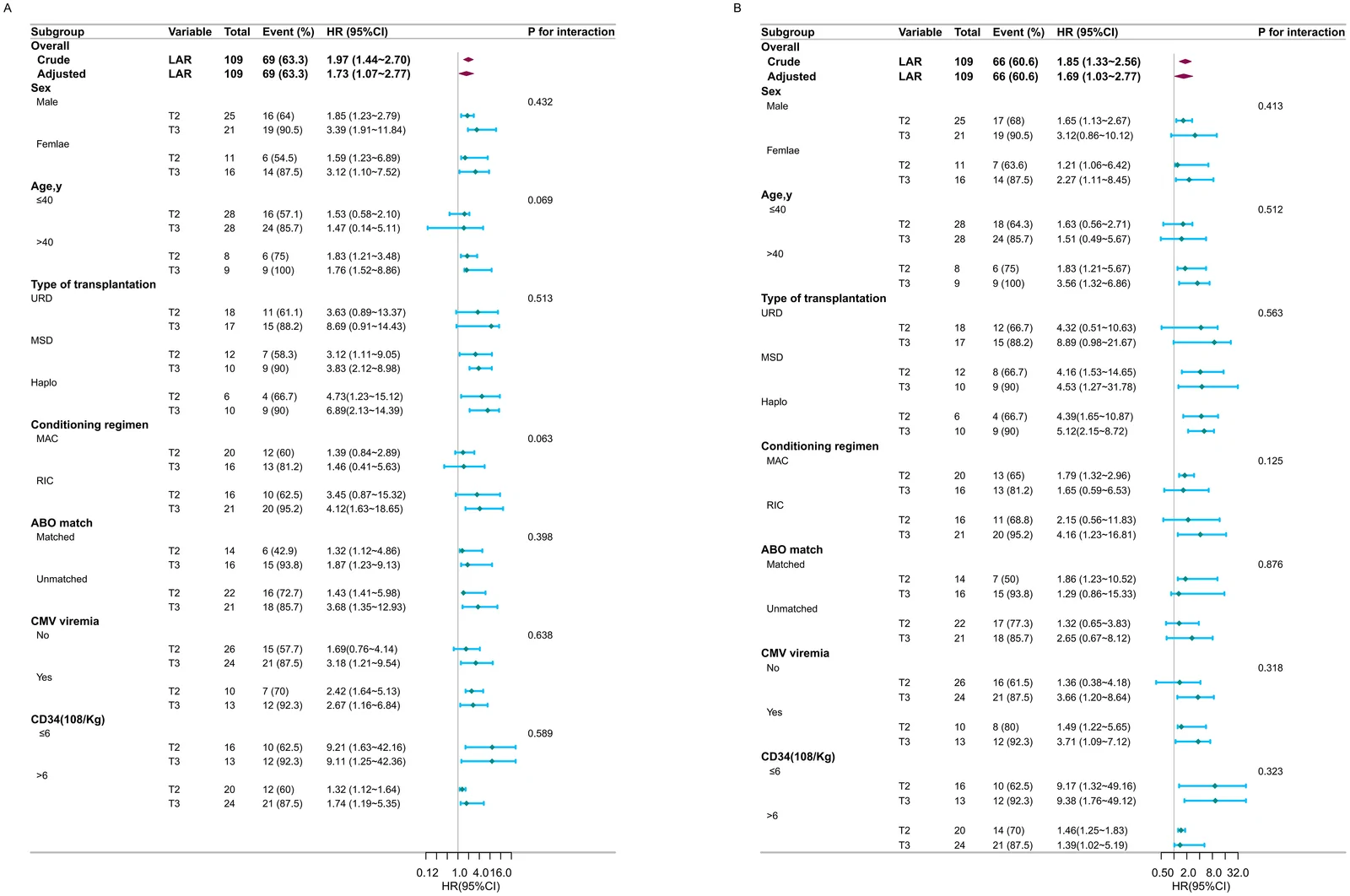
Forest plot ofsubgroup analyses for the association between LAR and all-cause mortality (ACM) and non-relapse morta lity(NRM). **(A)** Subgroup analysis of the association between LAR and ACM. **(B)** Subgroup analysis of theassociation between LAR and NRM. Adjusted hazard ratios (HR) and 95% confidence intervals (CI) from Coxproportionalhazards models (Model 3: adjusted for age, sex, transplantation covariates, and post-transplantclinical covariates) are shown across 7 predefined strata. The vertical dashed line indicates HR = 1.0. P valuesfor interaction are presented for each subgroup. No significant interactions were detected (all *P* for interaction >0.05).

### Sensitivity analyses

To assess whether early mortality events disproportionately influenced the primary findings, a sensitivity analysis was conducted that excluded 29 patients whose survival fell below 3 months (**Supplementary Table 3**). In this restricted cohort, the LAR–mortality association was substantially amplified. Continuous log-LAR demonstrated a stronger effect on ACM (Model 3: HR 4.14, 95% CI 1.79–9.63, *P* = 0.002) and NRM (Model 3: HR 2.54, 95% CI 1.18–5.46, *P* = 0.017). The graded tertile associations were also accentuated, with T3 conferring a more than four-fold increase in ACM (Model 3: HR 9.13, 95% CI 2.18–31.31, *P* = 0.001) and NRM risk (Model 3: HR 6.21, 95% CI 1.46–26.38, *P* = 0.013). These results confirm that the primary findings were not driven by early mortality events and indicate that LAR’s prognostic capacity is particularly pronounced among patients surviving beyond the early post-transplant period.

## Discussion

This retrospective cohort study investigated the association between the lactate dehydrogenase to albumin ratio (LAR) measured at the onset of aGVHD and mortality in patients undergoing allo-HSCT. Our principal findings demonstrate that elevated LAR is independently associated with increased risks of both all-cause mortality and non-relapse mortality, with the association persisting after comprehensive adjustment for demographic, transplantation-related, and post-transplant clinical covariates. Furthermore, the LAR exhibited a graded dose-response relationship with mortality endpoints, and the highest tertile conferred higher mortality risk compared with the lowest. These associations remained robust across extensive sensitivity and subgroup analyses.

The LAR’s prognostic significance in our cohort aligns with a growing body of literature supporting composite inflammation-nutrition biomarkers in transplantation outcomes. Composite ratios integrating inflammatory and nutritional parameters have demonstrated prognostic utility across diverse clinical contexts (32). The C-reactive protein-to-albumin ratio (CAR) has been shown to predict early post-transplant complications and survival in allo-HSCT recipients (24). Similarly, the blood urea nitrogen-to-albumin ratio (BAR) has emerged as a robust predictor of in-hospital mortality in critical illness and, more recently, has demonstrated prognostic value in aGVHD (33). Our finding that LAR-which similarly integrates a metabolic stress marker (LDH) with a nutritional reserve indicator (albumin)-independently predicts mortality extends this paradigm to the metabolic domain and suggests that composite biomarkers capturing distinct aspects of the host’s physiological state may offer superior prognostic performance compared with individual parameters.

The biological rationale underlying the LAR–mortality association is multifactorial. Elevated circulating LDH reflects not only increased cellular turnover and tissue damage–hallmarks of the inflammatory milieu characterizing aGVHD—but also serves as a surrogate for anaerobic glycolysis and the metabolic reprogramming associated with activated immune cells (34, 35). In the context of aGVHD, robust donor T-cell proliferation and target organ infiltration generate substantial metabolic demand, and LDH elevation may serve as an integrated readout of this process (36). Concurrently, hypoalbuminemia indicates both nutritional depletion and the presence of a systemic inflammatory state, as albumin synthesis is downregulated by pro-inflammatory cytokines, including interleukin-6 and tumor necrosis factor-α (23, 37). The convergence of these two dimensions–metabolic stress and nutritional-inflammatory depletion–within a single composite ratio may explain the LAR’s capacity to stratify mortality risk beyond that achievable by either parameter alone.T1 patients had earlier aGVHD onset (26 vs. ~76 days) yet showed the best survival, so time to onset is not a simple prognostic surrogate; after adjustment for time to onset (Model 2), LAR remained independently associated with mortality.

The dose-response characterization in our study revealed an inverted-L pattern for both mortality endpoints. The RCS analysis demonstrated a nonlinear relationship for both ACM (nonlinearity *P* = 0.006) and NRM (nonlinearity *P* = 0.009), with threshold analyses identifying breakpoints at LAR values of approximately 53 for ACM and 37 for NRM. In both cases, the hazard increased significantly below the threshold and plateaued above it, suggesting a ceiling effect wherein once a critical degree of metabolic disturbance is reached, further increments in LAR confer proportionally smaller increases in risk. This pattern is consistent with a saturation phenomenon observed with other composite biomarkers and may reflect the point at which metabolic stress becomes uniformly lethal irrespective of its magnitude (38). The clinical implication is that LAR may function as a particularly informative prognostic tool in the moderate-to-high risk range, with diminishing discriminatory capacity at extreme values.

This study possesses several strengths, including the comprehensive ascertainment of clinically relevant covariates, the systematic application of three hierarchical adjustment models, the rigorous characterization of dose-response relationships through RCS and threshold analyses, and the consistency of findings across multiple sensitivity and subgroup analyses. Nevertheless, several limitations merit consideration. First, as a retrospective, single-center study, it is inherently susceptible to selection bias and unmeasured confounding. Residual confounding by variables not captured in our dataset–such as the Hematopoietic Cell Transplantation Comorbidity Index (HCT-CI), detailed nutritional status, or longitudinal LAR trajectories–cannot be excluded. Second, the modest sample size (n=109) limits statistical power for subgroup analyses and precludes definitive assessment of interactions. Third, the single LAR measurement at aGVHD diagnosis does not capture the dynamic evolution of this biomarker in response to therapy; serial LAR measurements might yield additional prognostic information (39). Fourth, our cohort predominantly comprised patients with hematologic malignancies from a single ethnic background, potentially limiting generalizability to other populations or transplantation settings. Fifth, the optimal LAR threshold for risk stratification requires validation in an independent cohort before clinical implementation (40, 41). Machine learning and advanced computational algorithms have expanded the field of clinical outcome prediction (42, 43), including neural-network-based survival prediction in HSCT recipients (44, 45). These approaches demand large datasets and complex infrastructure, whereas simple biomarkers such as LAR offer interpretability, low cost, and immediate bedside applicability. Notwithstanding these limitations, our findings carry potential clinical implications. The LAR is derived from two routine laboratory tests–LDH and albumin–that are universally available, inexpensive, and obtained as part of standard clinical care in virtually all transplant centers. Its calculation requires no specialized platforms, additional cost, or incremental blood sampling, facilitating seamless integration into existing clinical workflows. If validated prospectively, the LAR could serve as a readily implementable tool for early risk stratification at aGVHD diagnosis, potentially guiding therapeutic intensity–for instance, identifying high-risk patients (LAR T3) who might benefit from intensified immunosuppression or early enrollment in clinical trials of novel agents. Subgroup and interaction analyses were exploratory and underpowered; their results are hypothesis-generating and require confirmation in larger cohorts. To limit overfitting, seven covariates were pre-specified based on clinical and biological rationale, without automated variable selection. With an events-per-variable ratio of ~10 and consistent sensitivity results, overfitting risk was limited, though not excluded; rigorous validation remains essential (46, 47) After adjustment for aGVHD grade, LAR remained independently associated with mortality. LAR and grade are nonetheless biologically related, and the incremental value of LAR beyond grading could not be quantified in this sample; LAR is best viewed as complementary to clinical grading.

Furthermore, the prognostic utility of LAR should be interpreted within the broader context of existing risk stratification frameworks. The EASIX score (Endothelial Activation and Stress Index), which incorporates LDH, creatinine, and platelet count, has been shown to predict endothelial complications and mortality after allo-HSCT (36, 48). Recent prospective multicenter validation has confirmed that an EASIX-pre cut-off of ≥3 identifies patients with a more than two-fold increased risk of non-relapse mortality, establishing EASIX as a clinically implementable tool (48). The MAGIC algorithm, integrating ST2 and REG3α, provides a validated “liquid biopsy” of gastrointestinal damage that predicts treatment response and NRM more accurately than clinical symptoms alone (13, 49, 50). Novel MAGIC composite scores that combine clinical severity with biomarker data have further improved prognostic discrimination (8, 49). The integration of LAR with these established tools—including aGVHD grade, MAGIC probability, and EASIX-may enable more precise risk discrimination than any single parameter alone. Future studies should explore whether combining LAR with these validated scores improves the accuracy of early mortality prediction and facilitates tailored therapeutic decision-making. Additionally, the dynamic trajectory of LAR during the course of aGVHD treatment warrants investigation, as longitudinal changes may provide more informative prognostic signals than a single baseline measurement, analogous to the serial monitoring paradigm established for MAGIC biomarkers (8, 13). Prospective multicenter studies with larger, more diverse cohorts are urgently needed to establish clinically applicable threshold values and to evaluate whether LAR-guided risk stratification translates into improved clinical outcomes.

## Conclusion

In this retrospective single-center cohort, LAR was independently associated with all-cause and non-relapse mortality. Given the modest sample size and the absence of formal discrimination and calibration assessment, these findings should be regarded as preliminary and await confirmation in larger, prospective, multicenter cohorts.

## Data Availability

The raw data supporting the conclusions of this article will be made available by the authors, without undue reservation.
