# Supplementary material for: Association of the lactate dehydrogenase-to-albumin ratio with all-cause and non-relapse mortality in patients with aGVHD after allogeneic HSCT: a single-center retrospective cohort study
Source: Front Immunol. 2026 Oct 9;17:1936503. doi: 10.3389/fimmu.2026.1936503 (PMC13690523; doi:10.3389/fimmu.2026.1936503)
Supplement: Supplementary file 1 [file Table1.docx]

| Item | n | Breakpoint/HR (95%CI) | P value |
| --- | --- | --- | --- |
| Estimate_Breakpoint_1 | 108 | 53.145 (52.102 ,54.189) | NA |
| Effect_Size_1 | 106 | 1.041 (1.003,1.08) | 0.0336 |
| Effect_Size_2 | 2 | 1.515 (0,Inf) | 0.9996 |
| Likelihood Ratio test | - | - | 0.468 |
