# Supplementary material for: Association of the lactate dehydrogenase-to-albumin ratio with all-cause and non-relapse mortality in patients with aGVHD after allogeneic HSCT: a single-center retrospective cohort study
Source: Front Immunol. 2026 Oct 9;17:1936503. doi: 10.3389/fimmu.2026.1936503 (PMC13690523; doi:10.3389/fimmu.2026.1936503)
Supplement: Supplementary file 2 [file Table2.docx]

| Item | n | Breakpoint/HR (95%CI) | P value |
| --- | --- | --- | --- |
| Estimate_Breakpoint_1 | 108 | 19.046 (18.479 ,19.612) | NA |
| Effect_Size_1 | 83 | 1.136 (1.055,1.223) | < 0.001 |
| Effect_Size_2 | 25 | 0.9975 (0.9693,1.0265) | 0.8627 |
| Likelihood Ratio test |  |  | 0.001 |
