# Supplementary material for: Association of the lactate dehydrogenase-to-albumin ratio with all-cause and non-relapse mortality in patients with aGVHD after allogeneic HSCT: a single-center retrospective cohort study
Source: Front Immunol. 2026 Oct 9;17:1936503. doi: 10.3389/fimmu.2026.1936503 (PMC13690523; doi:10.3389/fimmu.2026.1936503)
Supplement: Supplementary file 3 [file Table3.docx]

**Supplementary Table 3.** Association between LAR and mortality in patients with aGVHD

following allo-HSCT, excluding those with a survival time of less than 3 months

| Characteristic | Event,  n (%) | Crude model | |  | Model 1 | |  | Model 2 | |  | Model 3 | |
| --- | --- | --- | --- | --- | --- | --- | --- | --- | --- | --- | --- | --- |
|  |  | HR (95%CI) | *p*-Value |  | HR (95%CI) | *p*-Value |  | HR (95%CI) | *p*-Value |  | HR (95%CI) | *p*-Value |
| ACM |  |  |  |  |  |  |  |  |  |  |  |  |
| ln LAR | 42 (52.5) | 3.57 (2.32~5.50) | <0.001 |  | 3.95 (2.44~6.39) | <0.001 |  | 4.04 (2.25~7.27) | <0.001 |  | 4.14  (1.79~9.63) | 0.002 |
| LAR category |  |  |  |  |  |  |  |  |  |  |  |  |
| T1 | 6 (22.2) | 1(Ref) |  |  | 1(Ref) |  |  | 1(Ref) |  |  | 1(Ref) |  |
| T2 | 12 (46.2) | 2.23 (0.82~6.03) | 0.115 |  | 2.34 (0.83~6.58) | 0.107 |  | 1.85 (0.58~5.86) | 0.298 |  | 1.65 (0.83~6.97) | 0.315 |
| T3 | 24 (88.9) | 7.61 (3.05~18.96) | <0.001 |  | 7.99 (3.09~20.65) | <0.001 |  | 9.34 (2.99~29.17) | <0.001 |  | 9.13  (2.18~31.31) | 0.001 |
| Trend test |  |  | <0.001 |  |  | <0.001 |  |  | <0.001 |  |  | <0.001 |
| NRM |  |  |  |  |  |  |  |  |  |  |  |  |
| ln LAR | 40 (50) | 3.30 (2.12~5.13) | <0.001 |  | 3.83 (2.32~6.31) | <0.001 |  | 3.90 (2.12~7.17) | <0.001 |  | 2.54 (1.18~5.46) | 0.017 |
| LAR category |  |  |  |  |  |  |  |  |  |  |  |  |
| T1 | 7 (25) | 1(Ref) |  |  | 1(Ref) |  |  | 1(Ref) |  |  | 1(Ref) |  |
| T2 | 12 (44.4) | 2.26 (0.87~5.87) | 0.095 |  | 2.47 (0.92~6.66) | 0.074 |  | 2.58 (0.85~7.84) | 0.094 |  | 1.74 (0.47~6.46) | 0.409 |
| T3 | 21 (84) | 6.48 (2.68~15.66) | <0.001 |  | 7.31 (2.88~18.55) | <0.001 |  | 11.94 (3.52~40.52) | <0.001 |  | 6.21 (1.46~26.38) | 0.013 |
| Trend test |  |  | <0.001 |  |  | <0.001 |  |  | <0.001 |  |  | 0.007 |

LAR,Lactate Dehydrogenase to Albumin Ratio; T1, LAR ( 4.37-8.84); T2, LAR (8.84-16.02); T3, LAR(16.02-178.02); all-cause mortality; NRM, non-relapse mortality; aGVHD, acute graft-versus-host disease; allo-HSCT, allogeneic hematopoietic stem cell transplantation; HR, Hazard Ratio; CI, Confidence Interval; Ref, reference.

Model 1: Adjusted for Age and Sex；

Model2: Adjusted for Model1 and Indication for HSCT, Stem cell sources, Type of transplantation, Conditioning regimen, Days from transplantation to diagnosis, ABO match, MNC count, CD34^+^ cells count；

Model3: Adjusted for Model2 and Neutrophil engraftment time, CMV viremia, EBV viremia, White blood cells, Hemoglobin and Platelets, Total bilirubin, Creatinine, Pulmonary infection, Intestinal infection, Febrile neutropenia，aGVHD.
